# Emotion Reactivity Is Increased 4-6 Weeks Postpartum in Healthy Women: A Longitudinal fMRI Study

**DOI:** 10.1371/journal.pone.0128964

**Published:** 2015-06-10

**Authors:** Malin Gingnell, Elin Bannbers, Harmen Moes, Jonas Engman, Sara Sylvén, Alkistis Skalkidou, Kristiina Kask, Johan Wikström, Inger Sundström-Poromaa

**Affiliations:** 1 Department of Women’s and Children’s Health, Uppsala University, Uppsala, Sweden; 2 Department of Psychology, Uppsala University, Uppsala, Sweden; 3 Department of Radiology, Oncology and Radiation Science, Uppsala University, Uppsala, Sweden; University of Rennes-1, FRANCE

## Abstract

Marked endocrine alterations occur after delivery. Most women cope well with these changes, but the postpartum period is associated with an increased risk of depressive episodes. Previous studies of emotion processing have focused on maternal–infant bonding or postpartum depression (PPD), and longitudinal studies of the neural correlates of emotion processing throughout the postpartum period in healthy women are lacking. In this study, 13 women, without signs of post partum depression, underwent fMRI with an emotional face matching task and completed the MADRS-S, STAI-S, and EPDS within 48 h (early postpartum) and 4–6 weeks after delivery (late postpartum). Also, data from a previous study including 15 naturally cycling controls assessed in the luteal and follicular phase of the menstrual cycle was used. Women had lower reactivity in insula, middle frontal gyrus (MFG), and inferior frontal gyrus (IFG) in the early as compared to the late postpartum assessment. Insular reactivity was positively correlated with anxiety in the early postpartum period and with depressive symptoms late postpartum. Reactivity in insula and IFG were greater in postpartum women than in non-pregnant control subjects. Brain reactivity was not correlated with serum estradiol or progesterone levels. Increased reactivity in the insula, IFG, and MFG may reflect normal postpartum adaptation, but correlation with self-rated symptoms of depression and anxiety in these otherwise healthy postpartum women, may also suggest that these changes place susceptible women at increased risk of PPD. These findings contribute to our understanding of the neurobiological aspects of the postpartum period, which might shed light on the mechanisms underlying affective puerperal disorders, such as PPD.

## Introduction

A normal pregnancy and postpartum period are associated with major endocrine alterations which are adaptive changes in preparation for childbirth and nursing [1]. However some of these adaptations would be considered pathological if present in non-pregnant women. For instance, the postpartum period is characterized by suppression of estradiol and progesterone levels and the hypothalamic-pituitary-adrenal (HPA) axis [2], attenuated serotonergic activity [3–5], decreased cortical γ-butyric acid (GABA) concentrations [6], activation of the inflammatory response [7], and decreased responsivity of the autonomous defense system [8]. The hormonal changes across pregnancy and the puerperium have repeatedly been proposed as important risk factors for postpartum depression [9–10], indicating that emotion processing is affected by these adaptive changes.

Adequate emotion processing in the postpartum period is of immediate importance for maternal-infant bonding and involves attention, appropriate infant emotion recognition, reward/motivation, preoccupation of thought, and parental empathy [11–14]. Because of the relevance for short- and long-term offspring health, human imaging studies conducted in the postpartum period have focused predominantly on the neural correlates of postpartum depression [15–18], maternal behavior and attachment (using images of mothers’ own infants or infant cries as stimuli) [19–24], and changes in parental brain volume associated with maternal—infant bonding [25–26]. However, to enhance the understanding of the maternal brain, and to allow comparison with non-pregnant states, it is also important to investigate emotional processing in the postpartum period that is unrelated to motherhood. We previously reported that prefrontal brain activity during a response inhibition task was decreased throughout the first postpartum weeks in healthy women compared with non-pregnant control subjects [27] and using a task with affective images Rupp et al., [28] observed decreased amygdala reactivity in women 4–24 months post partum as compared to naturally cycling women. However, with a few exceptions [14], longitudinal studies of the neural correlates of emotion processing throughout the endocrine changes of the postpartum period in healthy women are scarce.

Emotion processing involves changes in reactivity within a network including the amygdala, insula and ACC as well as the prefrontal cortex [29, 30–33] and aberrant reactivity in this network have been reported to accompany symptoms of depression and anxiety [29]. Identification of the endocrine influence on emotion processing in the postpartum period among healthy women may serve as a valuable basis for future studies of postpartum depression and provide novel insights into how variations in ovarian steroid levels and the HPA axis affect such processing. An emotional face matching task based on work by Hariri and colleagues [34] has previously been used for assessment of emotional reactivity in relation to various hormonal states and the results suggest that ovarian steroid modulate reactivity in emotion processing areas such as the amygdala [35, 36] and insula [37]. A similar paradigm has also been used to display that reactivity in the dlPFC and connectivity dlPFC-amygdala is reduced in postpartum depression [16].

Amygdalar reactivity to various emotional stimuli (especially those with negative valence) has been reported to be increased during progesterone exposure, such as in the luteal phase of the menstrual cycle [35, 38, 39], and in response to single-dose progesterone administration [36]. In contrast, emotion-induced insular reactivity has been reported to be reduced in the presence of high estradiol and progesterone levels, [37, 41–42]. Mixed results, in terms of ovarian hormone responsiveness, have been reported for other emotion processing and regulatory areas, such as the OFC, ACC, mPFC, inferior frontal gyrus (IFG) and middle frontal gyrus (MFG) [39, 40, 41, 43, 44].

The aim of this longitudinal functional magnetic resonance imaging (fMRI) study was to examine the neural correlates of emotion processing in healthy postpartum women during a task that required matching of emotional faces. Participants were assessed at two time-point during the postpartum period; within 48 h of delivery when estradiol and progesterone levels still are relatively high; and 4–6 weeks postpartum when these hormones have reached their nadir. In addition, the postpartum women were compared with data from a previously published set of non-pregnant control subjects in the late luteal phase [30]. Based on their involvement in emotion processing [33, 40–43] and responsivity to changes in ovarian steroid levels [30–32, 34–39, 44] the bilateral amygdala, insula, ACC, IFG, and MFG were defined as regions of interest (ROIs). In comparison to the early postpartum period and naturally cycling controls, we predicted lower reactivity in the amygdala and higher reactivity in the insula during the late postpartum period, when ovarian steroid levels are suppressed. For ACC, IFG and MFG, results of previous studies are hitherto inconclusive and we did thus not have a directional hypothesis for these areas.

## Materials and Methods

### Participants

Twenty-six right-handed healthy postpartum women were recruited for this study from the maternity ward of the Department of Obstetrics and Gynecology, Uppsala University Hospital. Women aged 18–45 years with normal pregnancies, uncomplicated vaginal or Caesarean deliveries, and at least one night of sleep following delivery were included in the study. Exclusion criteria were postpartum complications; admission of infants to the neonatal intensive care unit; ongoing depression or anxiety disorders according to the Swedish version of the Mini International Neuropsychiatric Interview [45]; treatment with hormonal compounds or psychotropic drugs within 3 months prior to the study; and neurological disorders or previous brain trauma. Additional exclusion criteria, according to the guidelines of the hospital’s Center for Medical Imaging, were the use of a pacemaker, defibrillator, aneurysm clips, or any other metal implantation; visual impairment (>5 degrees myopia/hyperopia or profound astigmatism); and weight > 150 kg.

Sixteen healthy non-pregnant women who participated in our previous study [30] served as control subjects. They had self-reported regular menstrual cycles (25–31 days), did not use hormonal contraception, and were >1 year postpartum (or nulliparous) and not breastfeeding. Relevant exclusion criteria described above were also applied to these participants. All participants in the study were Caucasian.

All participants provided written informed consent prior to inclusion and the procedures were approved by the Regional Ethical Review Board, Uppsala, Sweden.

### Study design

Two fMRI sessions were scheduled for each participant in the postpartum group: within 48 h after delivery (early postpartum) and 4–6 weeks after delivery (late postpartum). For the naturally cycling controls, a session in the late luteal (postovulatory days 8–13) phase of the menstrual cycle was used for comparison with women early postpartum and a session in the mid-follicular phase (6–12 days post menstrual bleeding) for the comparison with women late postpartum (for further details, see [30]). The choice of comparison group was based on progesterone levels, as progesterone has greater influence on emotion processing than estradiol [46]. Scanning sessions for postpartum women could not be counterbalanced, for obvious reasons, but among healthy controls phase of entry was counterbalanced across phases. Blood samples for hormonal analyses were drawn approximately 20 min prior to each scanning session. Before each session, all participants were asked to complete two (control group) or three (postpartum group) questionnaires: the self-rated version of the Montgomery-Åsberg Depressive Rating Scale (MADRS-S) [47] to assess depressive symptoms during the previous 3 days, the state version of the Spielberger State-Trait Anxiety Inventory (STAI-S) [48] to evaluate state anxiety, and the Edinburgh Postnatal Depression Scale (EPDS; postpartum group) [49].

### Hormonal analyses

Serum progesterone and estradiol levels were analyzed by competitive immunometric electrochemical luminescence at the hospital’s Department of Medical Sciences using a Cobas e601 analyzer and Cobas Elecsys estradiol and progesterone reagent kits (Roche Diagnostics, Bromma, Sweden). The measurement intervals for progesterone and estradiol were 0.1–191 nmol/l and 18.4–15,781 pmol/l, respectively. The intra-assay coefficients of variation for progesterone were 2.2% at 2.4 nmol/l and 2.8% at 31.6 nmol/l, and those for estradiol were 6.8% at 85.5 pmol/l and 2.8% at 1640 pmol/l.

### fMRI paradigm

An emotion processing task based on that described by Hariri et al. [29] was used to activate the emotional areas of the brain. This paradigm comprises contrasting tasks involving emotion processing (three angry and fearful Ekman facial expressions) and simple sensorimotor control (three vertical or horizontal ellipses). Participants were instructed to select one of two images (displayed below the target image) displaying the same emotion or orientation as the target image (displayed at the top of the visual field) by pressing a button with the left or right index finger. Emotion and sensorimotor control task trials were presented in blocks of six, in which images were presented for 4 s, interspaced with a fixation cross (2 s for the sensorimotor control task and a randomly selected duration of 2, 4 or 6 s for the emotion task). The target facial expression or shape orientation differed among trials, with each emotion block containing an equal mix of emotions and sex of the individuals depicted. Accuracy and reaction times were registered for each trial. The paradigm consisted of four blocks of faces (total, 24 trials) and five blocks of shapes (total, 30 trials).

### fMRI data

MR imaging was performed using a whole-body scanner (Achieva 3T X; Philips Medical Systems, Best, The Netherlands) equipped with an eight-channel head coil. An anatomical T_1_-weighted reference dataset (voxel size, 0.8 × 1.0 × 2.0 mm^3^; 60 slices) was acquired at the beginning of each scanning session. During stimulus presentation, blood oxygen level—dependent (BOLD) imaging was performed using a single-shot echo planar imaging sequence (echo time/repetition time, 35/3000 ms; flip angle, 90°; acquisition matrix, 76 × 77; acquired voxel size, 3.0 × 3.0 × 3.0 mm^3^; 30 slices).

Participants underwent fMRI in supine position with the head lightly fixed with Velcro strips. During scanning, visual stimuli were presented through goggles mounted on the head coil (Visual System; NordicNeuroLab, Bergen, Norway). The stimulus paradigm was implemented using the E-prime commercial software package (Psychology Software Tools, Sharpsburg, PA, USA). To synchronize the paradigm with the scanner, trigger pulses from the scanner were fed to the paradigm-controlling PC through SyncBox (NordicNeuroLab).

DICOM images from the scanner were converted to NIfTI files using the MRicron freeware package (available at http://neuro.debian.net/pkgs/mricron.html). The data were analyzed in MatLab (MathWorks, Natick, MA, USA) using SPM 5 (available at http://www.fil.ion.ucl.ac.uk/spm/software/spm5/). BOLD images were realigned to create a mean image for each session, timed to the middle slice of each whole brain volume, co-registered with the individual’s anatomical scan, and normalized to Montreal Neurological Institute (MNI) space using parameters obtained from segmentation of the individual’s anatomical scan. Finally, smoothing was performed using an 8-mm Gaussian kernel (full width, half maximum). For each participant, the BOLD signal was regressed on the stimulus function (boxcar, onsets, and durations of facial stimuli and geometrical shapes) and six movement parameters obtained from the realignment step. The BOLD signal was convolved with the canonical hemodynamic response function provided by SPM. Contrast maps of reactivity to facial stimuli in contrast to that of geometrical shapes were produced for each individual. These maps were used for second-level random-effect group comparisons.

### Statistics

A mask was created of ROIs at the bilateral amygdala, insula, ACC, IFG, and MFG using anatomical automatic labeling definitions (amygdala, insula, and ACC) and Talairach Daemon labels (IFG and MFG) from the Wake Forest University PickAtlas [50–51]. Spatial localizations are reported in Talairach coordinates. Differences between early and late postpartum test sessions and between women post partum and healthy controls were analyzed with SPM using paired *t*-tests and an ANOVA followed by regular *t*-tests respectively, with a statistical threshold of *p* < 0.01 (small volume corrected) and an extent threshold of 10 contiguous voxels.

Comparisons of hormone levels, self-rated anxiety and depressive mood, and performance in the emotion-processing paradigm were performed by use of repeated measures ANOVA with group as between-subjects and time-point as within-subjects variables. Spearman rank correlation analyses were conducted to examine mean extracted ROI activity in arbitrary units. All analyses outside SPM were performed using SPSS software (version 20.0; SPSS Inc., Chicago, IL, USA). Values are expressed as means ± standard deviations, unless otherwise noted.

## Results

### Participants

A total of 46 women meeting initial inclusion criteria were asked to participate in the study, and 26 postpartum women agreed to participate. Out of those, eight could not be examined within 48 h after delivery due to lack of access to the fMRI equipment. One postpartum woman dropped out of the study after the first test session and three additional participants were excluded due to claustrophobic symptoms during scanning (*n* = 2) and nausea (*n* = 1). In addition, one postpartum woman was excluded from the fMRI analyses due to movement artifacts (peaks of movement > 3 mm on the *x*/*y*/*z* axis or >2° rotation). One of the 16 control subjects dropped out after the first scanning session. Thus, final analyses included 13 postpartum women and 15 naturally cycling control subjects. No significant differences in demographic or behavioral data were detected between excluded and remaining participants. Postpartum women participated in the first and second fMRI sessions 27 ± 10 h and 34 ± 5 days, respectively, after delivery.

### Demographic data

Table 1 provides demographic data for the study participants. No difference in age, number of previous pregnancies, body mass index, or educational level was detected between groups. A significantly larger proportion of postpartum women than control subjects was married or cohabiting. All postpartum women were breastfeeding. Eight (61.5%) postpartum women had vaginal deliveries and five (38.5%) underwent Caesarean section.

**Table 1:** Demographic and clinical data for the study population.

	Postpartum women(*n* = 13)	Non-pregnant control subjects(*n* = 15)	*p*
Age (years)	32.8 (4.2)	33.7 (8.4)	0.7
Pregnancies (*n*)	1.8 (1.2)	1.8 (2.1)	0.9
Body mass index (kg/m2)	24.0 (2.9)^a^	22.7 (3.7)	0.3
University education	12 (92.3%)	13 (86.7%)	0.6
Married or cohabiting	12 (92.3%)	6 (40.0%)	0.005^b^

Data are expressed as mean (standard deviation) or *n* (%).

^a^Self-reported pre-pregnancy values.

^b^Fisher’s exact test.

### Hormone levels

As expected, serum concentrations of estradiol and progesterone were higher in early than in late postpartum (estradiol, 1590 ± 709 *vs*. 120 ± 56 pmol/l; progesterone, 45.6 ± 37.7 *vs*. 0.8 ± 0.5 nmol/l; both *p* < 0.01). Estradiol and progesterone serum concentrations were significantly higher in early postpartum and significantly lower in late postpartum women than in naturally cycling control subjects in the luteal (estradiol, 414 ± 211 pmol; progesterone, 21.8 ± 13.1 nmol) and follicular (estradiol, 304 ± 150 pmol; progesterone, 3.4 ± 3.8 nmol) phase (all *p* < 0.05).

### Ratings of anxiety and depression

The ANOVA revealed a significant main effect of group for the MADRS-S scores, where postpartum women had higher scores than naturally cycling controls at both assessments. EPDS scores were higher in early than in late postpartum. There was no difference in state anxiety (STAI-S) between groups or test sessions (Table 2).

**Table 2:** Self-rated depression and anxiety and emotional paradigm performance.

	Postpartum women (*n* = 13)		Healthy controls (*n* = 15)		*F*-value
	Early postpartum m (SD)	Late postpartum m (SD)	Luteal phase m (SD)	Follicular phase m (SD)	Main effect of group^d^
*Mood and anxiety scores*					
EPDS	6.1 (3.9)	4.0 (2.5)^a^			
MADRS-S	10.6 (7.1)	7.3 (4.2)	3.2 (3.5)^b^	2.9 (2.9)^c^	17.29
STAI-S	32.8 (7.1)	29.0 (6.4)	28.6 (4.8)	28.6 (4.8)	2.47
*fMRI paradigm performance*					
Number of errors					
Faces	3.0 (3.4)	0.5 (0.7)	1.6 (1.3)	1.7 (1.8)	0.84
Shapes	0.5 (0.7)	0.5 (0.7)	1.1 (2.4)	0.4 (0.6)	0.34
Reaction time (s)					
Faces	2.2 (0.4)	2.1 (0.4)	2.0 (0.4)	2.0 (0.3)	0.89
Shapes	0.9 (0.1)	0.9 (0.1)	1.1 (2.4)	0.4 (0.6)	0.055

^a^ p < 0.05 in comparison with early postpartum, Wilcoxon Signed Ranks test.

^b^ p < 0.001 in comparison with early postpartum, Mann-Whitney U test.

^c^ p < 0.05 in comparison with late postpartum, Mann-Whitney U test.

^d^No group by time-point interactions were note in the ANOVA.

### fMRI data

**Fig 1:**
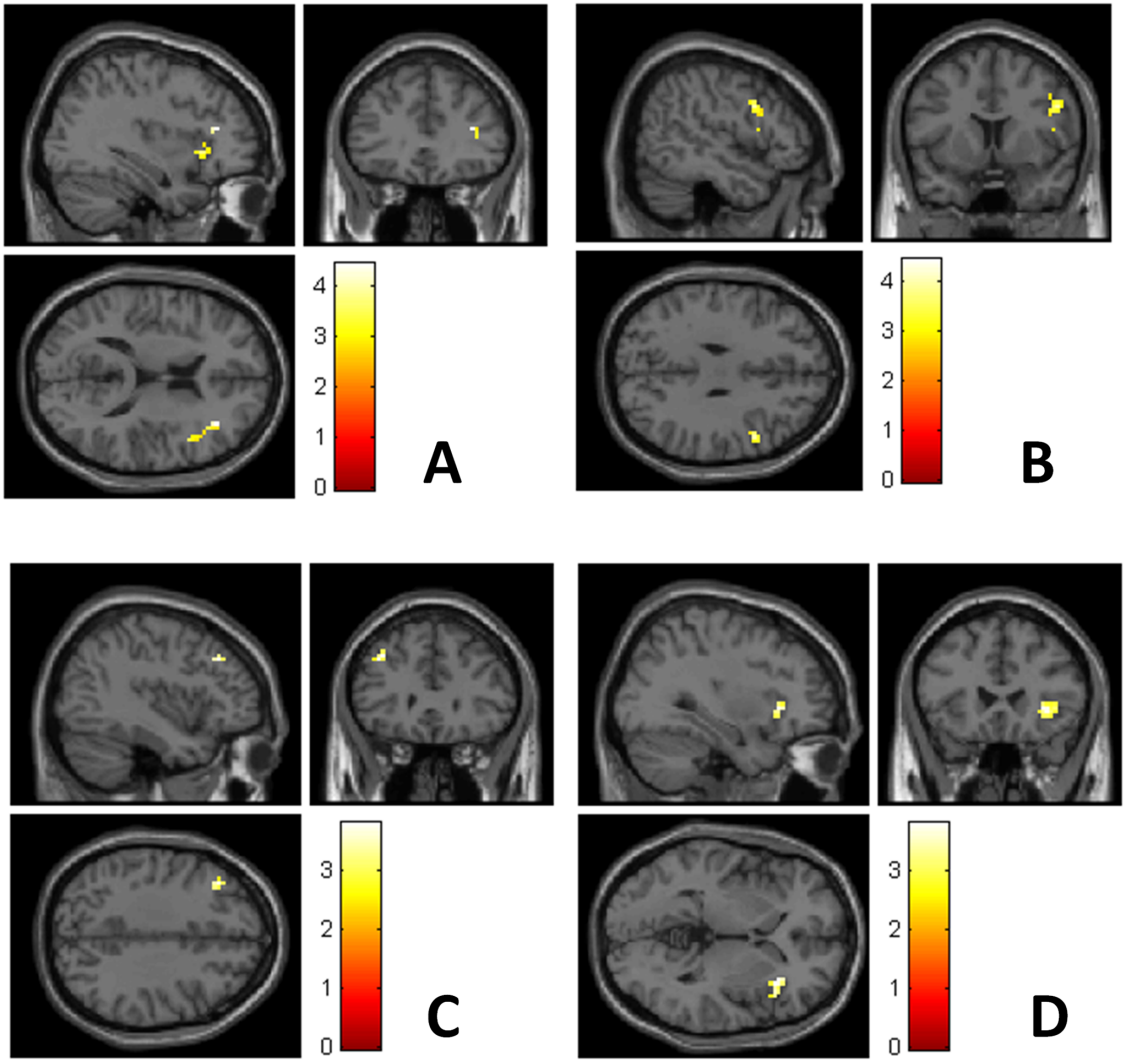
Reactivity in the right inferior frontal gyrus (A, B), left middle frontal gyrus (C) and right insula (D) during emotional stimulation was more pronounced in late postpartum than in early postpartum in 13 healthy newly delivered women. Brighter colors indicate higher T-scores. Early postpartum assessment was made within 48 hours of delivery, and late postpartum assessment within 4–6 weeks from delivery.

**Fig 2:**
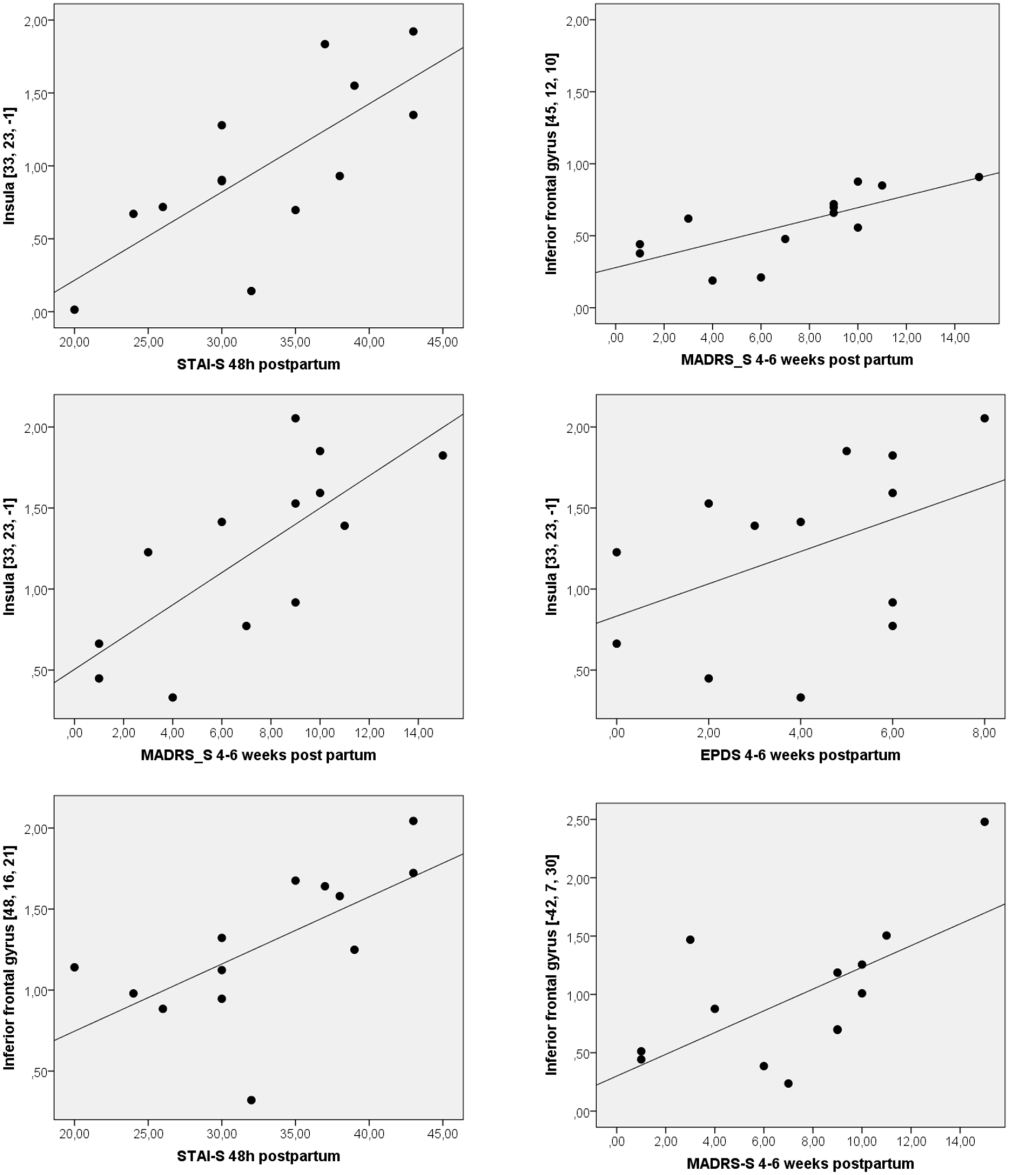
Positive correlations between reactivity in IFG and insula to scores on MADRS-S, STAI-S and EPDS in women early and late postpartum. Early postpartum assessment was made within 48 hours of delivery, and late postpartum assessment within 4–6 weeks from delivery.

Reactivity in the right insula, bilateral IFG and left MFG was lower in the early than in the late postpartum assessment (Table 3, Fig 1). At the early postpartum assessment reactivity in the IFG (BA 9) and the insula was positively correlated with state anxiety. At the late postpartum assessment reactivity in the IFG (BA 44 and 9) and the insula was positively correlated with self-rated depression, as assessed with the MADRS-S, and for insula also with a trend for the EPDS (Table 4, Fig 2). No correlation between brain reactivity and estradiol or progesterone serum concentration was observed at any of the postpartum time-points (S1 Table). There were no regions where reactivity was higher at the early postpartum than late post partum.

**Table 3:** Differences between the early and late postpartum period in blood oxygen level—dependent reactivity to emotional stimuli, N = 13.

					Talairach coordinates^a^			
Contrasts and regions of interest	BA	Hemisphere	Cluster size (mm^3^)	*Z* score	*x*	*y*	*z*	*p* ^b^
**early postpartum** ^**c**^ **> late postpartum**								
No significant cluster								
**late postpartum > early postpartum**								
Inferior frontal gyrus	46	R	2241	3.36	36	33	12	<0.001
Inferior frontal gyrus	44	R		3.52	45	12	10	<0.001
Insula	13	R		3.03	33	23	-1	0.001
Inferior frontal gyrus	9	R	945	3.07	50	10	27	0.001
Inferior frontal gyrus	9	R		2.82	48	16	21	0.001
Middle frontal gyrus	9	L	297	3.02	-39	31	32	0.001
Inferior frontal gyrus	9	L	351	3.03	-42	7	30	0.002
Middle frontal gyrus	9	L		2.57	-42	16	32	0.005

BA = Brodmann area, L = left, R = right.

^a^In Talairach stereotactic space.

^b^Corrected for multiple comparisons across the search volume of the region of interest, with an extent threshold cluster size ≥ 10.

^c^Early postpartum assessment was made within 48 hours of delivery, and late postpartum assessment within 4–6 weeks from delivery.

**Table 4:** Spearman rank correlations between blood oxygen level—dependent reactivity to emotional stimuli and self-reported anxiety and depression in early and late postpartum in 13 women.

	Early postpartum^b^			Late postpartum^b^		
	MADRS-S	STAIS-S	EPDS	MADRS-S	STAIS-S	EPDS
	*R*	*R*	*r*	*R*	*r*	*r*
Inferior frontal gyrus (BA 46)	0.39	0.37	0.086	0.48	-0.38	-0.26
Inferior frontal gyrus (BA 44)	-0.38	-0.14	0.025	0.80**	0.23	0.35
Insula (BA 13)	0.39	0.76**	0.24	0.72**	0.24	0.50^a^
Inferior frontal gyrus (BA 9)	-0.052	0.39	0.16	0.38	0.011	0.059
Inferior frontal gyrus (BA 9)	-0.091	0.71**	0.13	0.45	0.20	0.014
Middle frontal gyrus (BA 9)	-0.14	0.16	-0.23	0.48^a^	0.014	-0.25
Inferior frontal gyrus (BA 9)	-0.12	0.29	0.38	0.62*	0.21	0.034
Middle frontal gyrus (BA 9)	0.43	0.40	0.24	0.34	0.16	-0.18

MADRS-S = Montgomery Åsberg Depression Rating Scale—Self-rated version, STAI-S = Spielberger State-Trait Anxiety Inventory, EPDS = Edinburgh Postnatal Depression Scale.

*p < 0.05.

**p < 0.001.

^a^ *p* = 0.09, Spearman rank correlation (two-tailed).

^b^Early postpartum assessment was made within 48 hours of delivery, and late postpartum assessment within 4–6 weeks from delivery.

In the comparison with non-pregnant women, reactivity in the insula and IFG was found to be higher in postpartum women than in naturally cycling controls (Table 5). There was also a main effect of low hormone levels in insula, IFG and MFG (Table 5).

**Table 5:** Differences between postpartum women (n = 13) and naturally cycling control subjects (n = 15) in blood oxygen level—dependent reactivity to emotional stimuli.

					Tailarach coordinates^a^			
Contrasts and regions of interest	BA	Hemisphere	Cluster size (mm^3^)	*Z* score	*x*	*y*	*z*	*p* ^b^
**main effect of group** (women postpartum^c^ v.s. naturally cycling controls^d^)								
Inferior frontal gyrus	47	R	36	3.54	45	23	-1	< 0.001
Insula	13	L	30	2.94	-39	20	2	0.002
Insula	45	L		2.63	-27	27	7	0.004
**main effect of high/low hormone level** (early postpartum+luteal v.s. late postpartum+follicular)								
Precentral gyrus	6	L	61	3.27	-24	3	58	0.001
Precentral gyrus	6	L		3.19	-24	-3	53	0.001
Precentral gyrus	6	L		3.09	-27	-7	42	0.001
Precentral gyrus	6	R	10	2.86	30	-6	56	0.002
Inferior frontal gyrus	9	R	10	2.85	48	10	30	0.002
Insula	13	R	10	2.79	39	18	2	0.003
Inferior frontal gyrus	47	R		2.64	33	20	-4	0.004
**Interaction group x high/low hormone level**								
ACC	32	R	25	3.74	15	38	-2	< 0.001
Superior temporal	38	R	17	3.68	39	8	-13	< 0.001
Claustrum		L	15	3.26	-36	-8	6	0.001

BA = Brodmann area.

^a^In Talairach stereotactic space.

^b^Corrected for multiple comparisons across the search volume of the region of interest, with an extent threshold cluster size ≥ 10.

^c^Early postpartum assessment was made within 48 hours of delivery, and late postpartum assessment within 4–6 weeks from delivery.

^d^The healthy controls were randomly assigned to perform their first session in etiher the follicular or luteal phase.

At all sessions, higher reactivity was observed in response to faces than to shapes in large clusters including the bilateral amygdala, insula, IFG, and MFG (data not shown). Reaction times and the number of erroneous responses to emotional or sensorimotor control stimuli did not differ between the early and late postpartum assessment. Similarly, no difference between postpartum women and naturally cycling controls was detected (Table 2).

## Discussion

To our knowledge, this study represents one of the first attempts to address longitudinal changes in emotion-induced brain reactivity in healthy women during the postpartum period. Our findings indicate that reactivity in the IFG and insula were higher at 6 weeks postpartum than immediately following delivery. Notably, IFG and insular reactivity at 6 weeks postpartum were generally elevated as compared to healthy controls and were also correlated with depressive symptoms in these otherwise healthy, non-depressed postpartum women.

One of the most important distinguishing endocrine features of the postpartum period is the dramatic drop in estradiol and progesterone levels. Within a week from delivery (and the removal of the placenta), levels of ovarian steroid hormones drop from the 100-fold increase during pregnancy to levels that are in the postmenopausal range. Thus, insular reactivity increased over time in the postpartum period, i.e. during the shift from the high hormonal state of pregnancy to the low hormonal state of the postpartum period. Although the postpartum period is characterized by a multitude of hormonal and emotional changes in addition to a rapid decline in ovarian steroids, and no correlation between ovarian steroid levels and insular reactivity was noted, the increased insular reactivity in the late post partum period is consistent with those of previous studies suggesting a link between insular reactivity and changes in ovarian steroid levels [35, 41–42]. Previous studies have reported a relatively rapid increase in ovarian steroid levels to be accompanied by decreased insular reactivity to emotional stimuli [35]; the results of the present study show the opposite pattern, wherein the shift from high to low estradiol and progesterone levels was followed by an increase in insular reactivity. The transition from the supraphysiological hormone state of pregnancy to the postpartum period thus seems to affect insular reactivity to emotional stimuli.

Increased insular activation to emotional stimuli (or the anticipation thereof) may also underlie some depressive and anxious states [52–55]. While increased insular reactivity in our healthy postpartum women may represent an adaptation in emotion processing that supports effective parenting, it could also, hypothetically, contribute to the increased risk of depression observed postpartum. Even though participants in the present study reported sub-clinical levels of depressive symptoms, and previous studies have reported women with postpartum depression to have reduced emotion-induced left dorsomedial prefrontal cortex activation [16] and amygdala reactivity [16, 18, 56], areas that were not observed to be affected in the present study, the observed positive correlation between insular reactivity and depression scores at 4–6 weeks postpartum leaves preliminary support to the later theory. In addition, the observed early postpartum correlations with emotional reactivity and anxiety may reflect hormonal withdrawn manifested as “postpartum blues” with heightened emotionality, but may be important as postpartum blues is a strong risk factor for later development of depression [57] which may also account for the slightly higher depressive scores at the early assessment. In the late post partum period it is possible that this initial mood-response is abolished for the majority of women and thus also this correlation. However, especially for the insula there seem to be a shift from anxiety to depressive correlations at the late postpartum assessment. Even though the participants in the study were healthy, without any signs of PPD, we would argue that the correlations with depressive scores may indicate that the increase in insula and IFG reactivity in late postpartum could lay ground for development of postpartum depression in vulnerable women.

Delivery induces changes not only in hormonal levels but also increases maternal emotionality [48]. Most studies conducted in the postpartum period have examined maternal behavior and attachment by comparing mothers’ reactions to images and video clips of their infants with those to images of other infants or adult faces [20, 23]. Although the present study was not designed to examine changes in maternal behavior throughout the postpartum period, the observed changes in insular and IFG reactivity are in line with the findings of previous studies of maternal behavior [20–23] and may reflect an evolving maternal behavior and attachment process [20–23, 58], a theory which may be supported by the notion that postpartum women had higher insular reactivity as compared to naturally cycling controls, which was accentuated in the late postpartum period. However, as no measurement on maternal behavior or attachment was included in the study, these conclusions remain speculative.

A previous study on healthy women postpartum has reported of decreased amygdala reactivity in women postpartum as compared to naturally cycling women during watching of negative affective images [28]. This reduction was not observed in the present study, which may be due both to a different type of paradigm, but also to differences in time point for assessment and hormonal exposure. While the present study had a relatively narrow time-frame, 4–6 weeks postpartum, the study by Rupp et al., (2014) included women 1–6 months postpartum. There were also differences in hormonal exposure, whereas all participants in the present study had low levels of ovarian steroid hormones at the second scanning session, a substantial proportion of the postpartum women in the study by Rupp et al, (2014) where using hormonal contraceptives.

Using the same sample of women, we have previously observed that reduced reactivity in the IFG characterize the late postpartum period during an inhibitory task [27]. As the task used in the present study was not designed to address an explicit regulatory reactivity it is likely that the change in IFG reactivity observed in the present study is due to emotion exposure rather than emotional control. Nevertheless the inverse relationship with increased emotional reactivity in the late postpartum period and a decrease in inhibitory reactivity during the same period may suggest that the postpartum period could be characterized not only by increased demands for emotion processing, but also by a reduced need for cognitive control [27]. Further studies including paradigms with a more explicit emotion regulation may further clarify this relationship.

Although the aim of this study was to investigate emotion-induced brain activity that might be influenced by changes in ovarian steroid levels in the first postpartum weeks, we recognize that the different activation patterns in the postpartum period may also be related to HPA axis hyporesponsiveness [59], lingering dexamethasone non-suppression [60–61] or increased oxytocin levels [62], especially as all women postpartum were breastfeeding. Even though peripheral oxytocin levels are difficult to interpret, as they increase only during nursing [63] and do not cross the blood-brain barrier [64] the availability and analysis of plasma cortisol and oxytocin levels would have strengthened this study. However, a cross-sectional study by Rupp et al., [28] showed that the blunting effect of oxytocin on emotional brain reactivity may be less pronounced in women post partum than in healthy controls. Unfortunately, the present study also had insufficient power to determine whether the pattern of increased emotion-induced brain activity differed between primiparous and multiparous women, or between women with normal vaginal and Caesarean deliveries [24]. Future studies should address these issues, in addition to emotion responses to personally tailored stimuli. The present study is also limited by the small sample, especially for the attempt to estimate correlations between brain reactivity and ovarian steroid hormones, but it represents a hypothesis-generating attempt to describe the neuroanatomical correlates of emotion processing in the postpartum period in healthy, non-depressed women. The generalization of our findings to newly delivered women may be hampered by some characteristics of our study participants: these women were highly motivated, well educated, and had physically and psychologically uncomplicated deliveries and postpartum periods.

## Conclusions

In conclusion, this study demonstrated that emotion-induced insular and prefrontal reactivity increased throughout the first 4–6 postpartum weeks and that IFG and insular reactivity at the late postpartum assessment correlated positively with depression scores in healthy women. These findings contribute to our understanding of the neurobiological aspects of the postpartum period, which in turn might shed light on the mechanisms underlying maternal behavior, and possibly, affective disorders of the puerperium.

## Supporting Information

S1 Table Correlations amygdala-estradiol/progesterone. Correlations between amygdala reactivity during the emotional face matching task and estradiol/progesterone in women 48h and 4–6 weeks postpartum (n = 13).
(DOCX)

## References

[1] Grattan D. A mother's brain knows. J Neuroendocrinol. 2011;23: 1188–1189. 10.1111/j.1365-2826.2011.02175.x 22004569

[2] Meinlschmidt G, Martin C, Neumann ID, Heinrich M. Maternal cortisol in late pregnancy and hypothalamic-pituitary-adrenal reactivity to psychosocial stress postpartum in women. Stress. 2010;13: 163–171. 10.3109/10253890903128632 20214437

[3] Bailara KM, Henry C, Lestage J, Launay JM, Parrot F, Swendsen J, et al Decreased brain tryptophan availability as a partial determinant of post-partum blues. Psychoneuroendocrinology. 2006;31: 407–413. 16303256 10.1016/j.psyneuen.2005.10.001

[4] Doornbos B, Fekkes D, Tanke MA, de Jonge P, Korf J. Sequential serotonin and noradrenalin associated processes involved in postpartum blues. Prog Neuropsychopharmacol Biol Psychiatry. 2008;32: 1320–1325. 10.1016/j.pnpbp.2008.04.010 18502014

[5] Sacher J, Wilson AA, Houle S, Rusjan P, Hassan S, Bloomfield PM, et al Elevated brain monoamine oxidase A binding in the early postpartum period. Arch Gen Psychiatry. 2010;67: 468–474. 10.1001/archgenpsychiatry.2010.32 20439828

[6] Epperson CN, Gueorguieva R, Czarkowski KA, Stiklus S, Sellers E, et al Preliminary evidence of reduced occipital GABA concentrations in puerperal women: a 1H-MRS study. Psychopharmacology (Berl). 2006;186: 425–433. 16724188 10.1007/s00213-006-0313-7

[7] Kendall-Tackett K. A new paradigm for depression in new mothers: the central role of inflammation and how breastfeeding and anti-inflammatory treatments protect maternal mental health. Int Breastfeed J. 2007;2: 6 17397549 10.1186/1746-4358-2-6 PMC1855049

[8] Hellgren C, Bannbers E, Akerud H, Risbrough V, Poromaa IS. Decreased startle modulation during anticipation in the postpartum period in comparison to late pregnancy. Arch Womens Ment Health. 2012;15: 87–94. 10.1007/s00737-012-0261-7 22315106 PMC3305879

[9] O'Hara MW, McCabe JE. Postpartum depression: current status and future directions. Annu Rev Clin Psychol. 2013;9: 379–407. 10.1146/annurev-clinpsy-050212-185612 23394227

[10] Bloch M., Schmidt PJ, Danaceau M, Murphy J, Nieman L, Rubinow D. Effects of gonadal steroids in women with a history of postpartum depression. Am J Psychiatry. 2000;157: 924–930. 10831472 10.1176/appi.ajp.157.6.924

[11] Bartels A, Zeki S. The neural correlates of maternal and romantic love. Neuroimage. 2004;21: 1155–66. 15006682 10.1016/j.neuroimage.2003.11.003

[12] Leckman JF, Feldman R, Swain JE, Eicher V, Thompson N, Mayes LC. Primary Parental Preoccupation: Circuits, Genes, and the Crucial Role of the Environment. Journal of Neural Transmission 2004;111: 753–771. 15205997 10.1007/s00702-003-0067-x

[13] Kim P., Mayes LC, Feldman R, Leckman JF, Swain JE. Early Postpartum Parental Preoccupation and Positive Parenting Thoughts: Relationship with Parent-Infant Interaction. Journal of Infant Mental Health 2013;34: 104–116. 10.1002/imhj.21359 PMC4732877 26834300

[14] Swain JE, Kim P, Spicer J, Ho SS, Dayton CJ, Elmadih A, et al Approaching the Biology of Human Parental Attachment: Brain Imaging, Oxytocin and Coordinated Assessments of Mothers and Fathers. Brain Research. 2014;1580: 78–101. 10.1016/j.brainres.2014.03.007 24637261 PMC4157077

[15] Moses-Kolko EL, Wisner KL, Price JC, Berga SL, Drevets WC, Hanusa BH, et al Serotonin 1A receptor reductions in postpartum depression: a positron emission tomography study. Fertil Steril. 2008;89: 685–692. 17543959 10.1016/j.fertnstert.2007.03.059 PMC2410091

[16] Moses-Kolko EL, Perlman SB, Wisner KL, James J, Saul AT, Philips ML. Abnormally reduced dorsomedial prefrontal cortical activity and effective connectivity with amygdala in response to negative emotional faces in postpartum depression. Am J Psychiatry. 2010;167: 1373–1380. 10.1176/appi.ajp.2010.09081235 20843875 PMC3293151

[17] Moses-Kolko EL, Fraser D, Wisner KL, James JA, Saul AT, Fiez JA, et al Rapid habituation of ventral striatal response to reward receipt in postpartum depression. Biol Psychiatry. 2011;70: 395–399. 10.1016/j.biopsych.2011.02.021 21507385 PMC3454468

[18] Silverman ME, Loudon H, Liu X, Mauro C, Leiter G, Goldstein MA. The neural processing of negative emotion postpartum: a preliminary study of amygdala function in postpartum depression. Arch Womens Ment Health. 2011;14: 355–359. 10.1007/s00737-011-0226-2 21713456

[19] Seifritz E, Esposito F, Neuhoff JG, Lüthi A, Mustovic H, Dammann G, et al Differential sex-independent amygdala response to infant crying and laughing in parents versus nonparents. Biological Psychiatry. 2003;54: 1367–1375. 14675800 10.1016/s0006-3223(03)00697-8

[20] Leibenluft E, Gobbini MI, Harrison T, Haxby JV. Mothers' neural activation in response to pictures of their children and other children. Biol Psychiatry. 2004;56: 225–232. 15312809 10.1016/j.biopsych.2004.05.017

[21] Noriuchi M, Kikuchi YSenoo A. The functional neuroanatomy of maternal love: mother's response to infant's attachment behaviors. Biol Psychiatry. 2008;63: 415–423. 17686467 10.1016/j.biopsych.2007.05.018

[22] Swain JE, Tasgin E, Mayes LC, Feldman R, constable RT, Leckman JF. Maternal brain response to own baby-cry is affected by cesarean section delivery. J Child Psychol Psychiatry. 2008:49: 1042–1052. 10.1111/j.1469-7610.2008.01963.x 18771508 PMC3246837

[23] Lenzi D, Trentini C, Pantano P, Macaluso E, Iacoboni M, Lenzi GL, et al Neural basis of maternal communication and emotional expression processing during infant preverbal stage. Cereb Cortex. 2009;19: 1124–1133. 10.1093/cercor/bhn153 18787229

[24] Kim P, Feldman R, Leckman JF, Mayes LC, Swain JE. Breastfeeding, Brain Activation to Own Infant Cry, and Maternal Sensitivity. Journal of Child Psychology and Psychiatry 2011;52: 907–915. 10.1111/j.1469-7610.2011.02406.x 21501165 PMC3134570

[25] Kim P, Leckman JF, Mayes LC, Feldman R, Wang X, Swain JE. The plasticity of human maternal brain: longitudinal changes in brain anatomy during the early postpartum period. Behav Neurosci. 2010;124: 695–700. 10.1037/a0020884 20939669 PMC4318549

[26] Kim P, Rigo P, Mayes LC, Feldman R, Leckman JF, Swain JE. Neural plasticity in fathers of human infants. Social Neuroscience 2014;9: 522–535. 10.1080/17470919.2014.933713 24958358 PMC4144350

[27] Bannbers E, Gingnell M, Engman J, Morell A, Sylvén S, Skalkidou A, et al Prefrontal activity during response inhibition decreases over time in the postpartum period. Behav Brain Res. 2013;241: 132–138. 10.1016/j.bbr.2012.12.003 23238040

[28] Rupp HA, James TW, Ketterson ED, Sengelaub DR, Ditzen B, Heiman JR. Amygdala response to negative images in postpartum vs nulliparous women and intranasal oxytocin. Soc Cogn Affect Neurosci. 2014;9: 48–54. 10.1093/scan/nss100 22956670 PMC3871727

[29] Shin LM, Liberzon I. The neurocircuitry of fear, stress, and anxiety disorders. Neuropsychopharmacology. 2010;35: 169–191. 10.1038/npp.2009.83 19625997 PMC3055419

[30] Davidsson R, Putnam K, Larson C. Dysfunction in the neural circuitry of emotion regulation—a possible prelude to violence. Science. 2000;258: 591–594. 10.1126/science.289.5479.591 10915615

[31] Bush G, Luu P, Posner MI. Cognitive and emotional influences in anterior cingulate cortex. Trends in Cognition & Science. 2000;4: 215–222. 10.1016/s1364-6613(00)01483-2 10827444

[32] Fusar-Poli P, Placentino A, Carletti F, Landi P, Allen P, Surguladze S, et al Functional atlas of emotional faces processing: a voxel-based meta-analysis of 105 functional magnetic resonance imaging studies. Journal of Psychiatry & Neuroscience. 2009;34: 418–443. 10.3233/JAD-131544 19949718 PMC2783433

[33] Pessoa L, Adolphs R. Emotion processing and the amygdala: from a 'low road' to 'many roads' of evaluating biological significance. Nat Rev Neuroscience. 2010;11: 773–783. 20959860 10.1038/nrn2920 PMC3025529

[34] Hariri AR, Mattay VS, Tessitore A, Kolachana B, Fera F, Goldman D, et al Serotonin transporter genetic variation and the response of the human amygdala. Science. 2002;297: 400–403. 12130784 10.1126/science.1071829

[35] Gingnell M, Morell A, Bannbers E, Wikström J, Sundström Poromaa I. Menstrual cycle effects on amygdala reactivity to emotional stimulation in premenstrual dysphoric disorder. Horm Behav. 2012;62: 400–406. 10.1016/j.yhbeh.2012.07.005 22814368

[36] van Wingen GA, van Broekhoven F, Verkes RJ, Petersson KM, Backstrom T, Buitelaar TK, et al Progesterone selectively increases amygdala reactivity in women. Molecular Psychiatry. 2008;13: 325–333. 17579609 10.1038/sj.mp.4002030

[37] Gingnell M, Engman J, Frick A, Moby L, Wikström J, Fredrikson M, et al Oral contraceptive use changes brain activity and mood in women with previous negative affect on the pill-A double-blinded, placebo-controlled randomized trial of a levonorgestrel-containing combined oral contraceptive. Psychoneuroendocrinology. 2013;38: 1133–1144. 10.1016/j.psyneuen.2012.11.006 23219471

[38] Andreano JM, Cahill L. Menstrual cycle modulation of medial temporal activity evoked by negative emotion. Neuroimage. 2010;53: 1286–1293. 10.1016/j.neuroimage.2010.07.011 20637290 PMC3376005

[39] Bayer J, Schultz H, Gamer M, Sommer T. Menstrual-cycle dependent fluctuations in ovarian hormones affect emotional memory. Neurobiol Learn Mem. 2014;110: 55–63. 10.1016/j.nlm.2014.01.017 24492058

[40] Protopopescu X, Pan H, Altemus M, Tuescher O, Polanecsky M, McEwen B, et al Orbitofrontal cortex activity related to emotional processing changes across the menstrual cycle. Proc Natl Acad Sci U S A. 2005;102:16060–16065. 16247013 10.1073/pnas.0502818102 PMC1276043

[41] Love T, Smith YR, Persad CC, Tkaczyk A, Zubieta JK. Short-term hormone treatment modulates emotion response circuitry in postmenopausal women. Fertil Steril. 2010;93: 1929–1937. 10.1016/j.fertnstert.2008.12.056 19243753 PMC2894029

[42] Shafir T, Love T, Berent-Spillson A, Persad CC, Wang H, Reame NK, et al Postmenopausal hormone use impact on emotion processing circuitry. Behav Brain Res. 2012;226: 147–153. 10.1016/j.bbr.2011.09.012 21930160 PMC3201705

[43] Dreher JC, Schmidt PJ, Kohn P, Furman D, Rubinow D, Berman KF. Menstrual cycle phase modulates reward-related neural function in women. Proceedings of the National Academy of Sciences U S A. 2007;104: 2465–2470. 17267613 10.1073/pnas.0605569104 PMC1892961

[44] van Wingen GA, Ossewaarde L, Backstrom T, Hermans EJ, Fernandez G. Gonadal hormone regulation of the emotion circuitry in humans. Neuroscience. 2011;191: 38–45. 10.1016/j.neuroscience.2011.04.042 21540080

[45] Sheehan DV, Lecrubier Y, Sheehan KH, Amorim P, Janavs J, Weiller E, et al The Mini-International Neuropsychiatric Interview (M.I.N.I.): the development and validation of a structured diagnostic psychiatric interview for DSM-IV and ICD-10. J Clin Psychiatry. 1998;59 Suppl 20:22–33;quiz 34–57. 9881538

[46] Sundström Poromaa I, Gingnell M. Menstrual cycle influence on cognitive function and emotion processing-from a reproductive perspective.Front Neurosci. 2014;8: 380 10.3389/fnins.2014.00380 25505380 PMC4241821

[47] Montgomery S, Åsberg M. A new depression scale designed to be sensitive to change. Br J Psychiatry. 1979;134: 382–389. 444788 10.1192/bjp.134.4.382

[48] Spielberger CD, Gorusch RL, Lushene R. Manual for the State-Trait Anxiety (STAI Form Y), Consulting Psychologist Press, Palo Alto, CA, 1983

[49] Cox JL, Holden JM, Sagovsky R. Detection of postnatal depression: Development of the 10-item Edinburgh Postnatal Depression Scale. British Journal of Psychiatry. 1987;150: 782–786. 3651732 10.1192/bjp.150.6.782

[50] Lancaster J, Summerlin J, Rainey L, Freitas C, Fox P. The Talairach Daemon, A Database Server for Talairach Atlas Labels. *Neuroimage* 1997;5(4, Part 2 of 4 Parts): S633 Academic Press.

[51] Maldjian JA, Laurienti PJ, Kraft RA, Burdette JH. An automated method for neuroanatomic and cytoarchitectonic atlas-based interrogation of fMRI data sets. Neuroimage. 2003;19: 1233–1239. 12880848 10.1016/s1053-8119(03)00169-1

[52] Mitterschiffthaler MT, Kumari V, Malhi GS, Brown RG, Giampietro VP, Brammer MJ, et al Neural response to pleasant stimuli in anhedonia: an fMRI study. Neuroreport. 2003;14: 177–182. 12598724 10.1097/00001756-200302100-00003

[53] Simmons A, Strigo I, Matthews SC, Paulus MP, Stein MB. Anticipation of aversive visual stimuli is associated with increased insula activation in anxiety-prone subjects. Biol Psychiatry. 2006;60: 402–409. 16919527 10.1016/j.biopsych.2006.04.038

[54] Stein MB, Simmons AN, Feinstein JS, Paulus MP. Increased amygdala and insula activation during emotion processing in anxiety-prone subjects. Am J Psychiatry. 2007;164: 318–327. 17267796 10.1176/ajp.2007.164.2.318

[55] Strigo IA, Simmons AN, Matthews SC, Craig AD, Paulus MP. Association of major depressive disorder with altered functional brain response during anticipation and processing of heat pain. Arch Gen Psychiatry. 2008;65: 1275–1284. 10.1001/archpsyc.65.11.1275 18981339 PMC2702160

[56] Silverman ME, Loudon H, Safier M, Protopopescu X, Leiter G, Liu X, et al Neural dysfunction in postpartum depression: an fMRI pilot study. CNS Spectr. 2007;12: 853–862. 17984858 10.1017/s1092852900015595

[57] Reck C, Stehle E, Reinig K, Mundt C. Maternity blues as a predictor of DSM-IV depression and anxiety disorders in the first three months postpartum. J Affect Disord. 2009;113: 77–87. 10.1016/j.jad.2008.05.003 18573539

[58] Newman LK, Harris MAllen J. Neurobiological basis of parenting disturbance. Aust N Z J Psychiatry. 2011;45: 109–122. 10.3109/00048674.2010.527821 20977304

[59] Altemus M, Deuster PA, Galliven E, Carter CS, Gold PW. Suppression of hypothalmic-pituitary-adrenal axis responses to stress in lactating women. J Clin Endocrinol Metab. 1995;80: 2954–2959. 7559880 10.1210/jcem.80.10.7559880

[60] Smith R, Owens PC, Brinsmead MW, Singh B, Hall C. The nonsuppressibility of plasma cortisol persists after pregnancy. Horm Metab Res. 1997;19: 41–42. 10.1055/s-2007-1011733 3557282

[61] Maes M, Claes M, Schotte C, Delbeke L, Jacquemyn Y, Verkerk R, et al Disturbances in dexamethasone suppression test and lower availability of L-tryptophan and tyrosine in early puerperium and in women under contraceptive therapy. J Psychosom Res. 1992;36: 191–197. 1560430 10.1016/0022-3999(92)90028-z

[62] Nissen E, Lilja G, Widstrom AM, Ulvnas-Moberg K. Elevation of oxytocin levels early post partum in women. Acta Obstet Gynecol Scand. 1995;74: 530–533. 7618451 10.3109/00016349509024384

[63] Dawood MY, Khan-Dawood FS, Wahi RS, Fuchs F. Oxytocin release and plasma anterior pituitary and gonadal hormones in women during lactation. J Clin Endocrinol Metab. 1981;52: 678–683. 6782115 10.1210/jcem-52-4-678

[64] Altemus M, Fong J, Yang R, Damast S, Luine V, Ferguson D. Changes in cerebrospinal fluid neurochemistry during pregnancy. Biol Psychiatry. 2004;56: 386–392. 15364035 10.1016/j.biopsych.2004.06.002

