# Supplementary material for: Emotion Reactivity Is Increased 4-6 Weeks Postpartum in Healthy Women: A Longitudinal fMRI Study
Source: PLoS One. 2015 Jun 10;10(6):e0128964. doi: 10.1371/journal.pone.0128964 (PMC4465482; doi:10.1371/journal.pone.0128964)
Supplement: S1 Table — Correlations between amygdala reactivity during the emotional face matching task and estradiol/progesterone in women 48h and 4–6 weeks postpartum (n = 13). (DOCX) [file pone.0128964.s001.docx]

| **Supplemental Table 1** Correlations between reactivity during an emotional face matching task and estradiol/progesterone in women 48h and 4-6 weeks postpartum (n=13). | | | | | | | | |
| --- | --- | --- | --- | --- | --- | --- | --- | --- |
|  | IFG1_46 | IFG1_44 | insula | IFG2_9 | IFG3_9 | MFG1 | IFG4 | MFG2 |
| **Early postpartum** | |  |  |  |  |  |  |  |
| Estradiol | -0.450 | -0.433 | -0.067 | 0.100 | -0.400 | 0.183 | -0.100 | -0.083 |
| Progesterone | -0.276 | -0.201 | 0.184 | -0.017 | -0.234 | -0.343 | 0.402 | 0.109 |
|  |  |  |  |  |  |  |  |  |
| **Late postpartum** | |  |  |  |  |  |  |  |
| Estradiol | -0.560^a^ | -0.500^b^ | -0.401 | 0.209 | -0.121 | 0.192 | 0.038 | -0.060 |
| Progesterone | 0.456 | -0.022 | 0.379 | 0.335 | 0.385 | 0.055 | 0.066 | -0.121 |

^a)^ p=0.05 ^b)^ p = 0.082
